# Bronchiectasis

**Published:** 1908-07-25

**Authors:** 


### Diseases of Children.

### BRONCHIECTASIS.

It is not very uncommon for a child to come under observation for a troublesome cough, of many months' duration, and perhaps associated with vomiting. On inquiry into the previous history one generally finds that the child has had attacks of bronchitis or broncho-pneumonia, frequently as a complication or sequel of measles or pertussis. These children are anæmic, ill-nourished, and present on physical examination marked signs of consolidation in some region of the chest. The diagnosis then rests between chronic pneumonia, fibrosis of the lung, tuberculosis, new growth, empyema, and bronchiectasis. A study of the physical signs shows how difficult it may be to make an accurate diagnosis. In one instance an attack of pneumonia, with slight pleurisy, terminates in the usual way by crisis; in another the temperature only falls gradually to about the normal level. But the physical signs do not clear up. There remain dulness, râles, and patchy bronchial breathing, with undue feebleness of air entry over the affected lung. It is this feebleness of air entry which should attract attention, especially in relation to the bronchial breathing. Alone, it is suggestive most of empyema. The bronchial breathing is, however, very variable, not constant in quality as usual in empyema, for at one time it may be feeble, distant, or absent, and at another extremely loud. This difference depends on whether the dilated bronchi or bronchioles are full or empty. Sometimes the breathing is even cavernous. In course of time the fibrosed lung may contract and cause a compensatory contraction of the chest, with displacement of the heart to the affected side. Empyema in the early stages may cause displacement of the heart to the unaffected side, but in the rare instances of re-absorption in late stages it may produce a similar effect to that due to fibrosis of the lung and bronchiectasis.

In babies there is little further assistance to be got from physical examination in early stages. Later on, as a result of decomposition of retained secretion in the cavities, the breath becomes offensive, and fœtid sputa may be brought up in the attacks of vomiting induced by the violent spasmodic attacks of coughing. Such vomiting may occur once or twice a day, generally in the early morning. Older children can be taught to expectorate, and the sputum should be examined for tubercle bacilli. Hæmoptysis sometimes occurs. Hence an erroneous diagnosis of phthisis is apt to be arrived at on account of the association of symptoms, namely, hæmoptysis, wasting, cough, expectoration, anæmia, and signs of a cavity in the lung. The fact that the physical signs are more often situated at the base in bronchiectasis and at the apex in tuberculosis is of practically no assistance, for in children both parts of the lung are liable to be affected with either disease. At present I have a boy under my care who has had recurrent attacks of broncho-pneumonia, chiefly affecting the upper lobe of the left lung, for nearly six months. He still presents signs of consolidation of the upper lobe, dulness, loud bronchial breathing, bronchophony, and a few râles. Such a state of the lung is very suggestive of tuberculosis, but on the whole I believe there is nothing more than fibrosis secondary to chronic broncho-pneumonia and possibly some dilatation of the bronchial tubes. Cases of this nature have been under my observation for several years, and have eventually cleared up entirely. Another frequent symptom is clubbing of the fingers and toes.

Hence the chief diagnostic features are anæmia, malnutrition, clubbing of the extremities, offensive breath and sputa, and physical signs of consolidation or patchy excavation, notably varying bronchial breathing and feeble breath sounds. Fever is commonly slight or absent until towards the end, when it may become hectic in type from secondary septic infection. The nutrition is impaired in most cases, but under careful treatment rapidly improves, although there may be little or no improvement in the physical signs. Thus a boy, aged ten years, put on 71 oz. in weight in six weeks, in spite of hæmoptysis and offensive expectoration. The chest was contracted and the physical signs characteristic, with no fever. After some months' treatment and a visit to the seaside, the chest showed no abnormal physical signs, except a little impairment of resonance at the base and deficient air entry. It had fully expanded, and the boy looked the picture of health. Such a good result as this is unfortunately rare. In infants the affection is liable to be complicated by rickets, with the result that the yielding chest wall becomes markedly pigeon-breasted and the mechanism of respiration is still further interfered with. Consequently the prognosis is worse.

Chronic cases can be arrested, improved by treatment, and may get well. The prognosis varies with the extent. Many are progressive and incurable and last for years. The indications for treatment are to empty the cavities, relieve fœtor, encourage full expansion of the lungs, prevent attacks of pulmonary catarrh, and maintain the general health. Posture assists in emptying the cavities and may be aided mechanically, *e.g.* by inversion or by leaning over the side of the bed with the hands on the floor, on waking in the morning and three times a day; compression of the abdomen during expiration; holding up the child by the legs and compression of the chest. Expectorant mixtures containing pot. iod. are useful and may be followed every fourth day by an emetic of vin. ipecac. Emptying the cavities prevents fœtor. It can be further relieved by sprays or inhalations of terebene, ol. pini sylvest., eucalyptus, creosote, thymol, etc.; creosote vapour baths; intra-tracheal injections of izal 10 per cent. in glycerin, or guaiacol 2, menthol 10, olive oil 88 parts; by drugs internally, given in capsules after food, such as creosote, oil of garlic ½ ♏, syr. allii U.S.P., syr. picis liq. U.S.P., or ol. tereben. Codliver oil, syr. fer. iod., hypophosphites, quinine, and arsenic may also be given. Food should be liberal and nutritious; hygiene, that suitable for tuberculosis; and the climate warm, dry, and equable, such as Jamaica, South California, South Africa, West Indies, Mediterranean. Operative treatment is not advisable.

